# Guidelines for collecting vouchers and tissues intended for genomic work (Smithsonian Institution): Botany Best Practices

**DOI:** 10.3897/BDJ.5.e11625

**Published:** 2017-01-30

**Authors:** Vicki A Funk, Morgan Gostel, Amanda Devine, Carol L Kelloff, Kenneth Wurdack, Chris Tuccinardi, Aleks Radosavljevic, Melinda Peters, Jonathan Coddington

**Affiliations:** 1 Department of Botany, National Museum of Natural History, Smithsonian Institution, Washington DC, United States of America; 2 Global Genome Initiative (GGI), National Museum of Natural History, Smithsonian Institution, Washington DC, United States of America

**Keywords:** genomic tissue, plant collecting, cryopreservation, biorepository, vouchers, botanical gardens, arboreta, biological collections, herbarium specimens

## About this Training Manual

The Smithsonian Institution’s Department of Botany (includes the US National Herbarium; US) recently embarked on a project to voucher and collect genome quality tissue samples from the plants growing in mid-Atlantic botanical gardens, greenhouses, and arboreta (GGI-Gardens). This effort, funded by the Global Genome Initiative (GGI), is part of the Global Genome Biodiversity Network (GGBN; Seberg et al. 2016). The rise of GGI-Gardens happened in conjunction with the recent broad-scale increase in the department for collecting samples for genomic research. As a result, we have developed some ‘best practices’ that we present here. Although, some of the topics are specific to US we hope they will be useful to a broader audience as a starting point for other ‘Best Practices’ documents. There is a Word file available for those who wish to amend this document for their own purposes. This manual began as a workflow document (Gostel et al. 2016) and developed into its current form as a result of our efforts to establish field collection standards for botanical collections in the Department of Botany (Smithsonian Institution) and associated tissue samples that are intended for incorporation into the National Museum of Natural History’s (NMNH) Biorepository. We include information on collecting voucher specimens, collecting plant tissue intended for genomic analysis, how to manage these collections, and how to incorporate the data into our database management systems (EMu for the vouchers and FreezerPro for the Biorepository). We hope it will be useful for a variety of botanists but especially those who know how to collect plants and want to collect tissue samples that will be useful for genomic research, and those who are skilled in lab work and want to know how to properly voucher their tissue collections. There are many articles and manuals on managing herbaria and on how to collect herbarium specimens (Applequist and Campbell 2014, Davis 2011, Davis 1961, Fosberg and Sachet 1965, Hyland 1972, Jain and Mudgal 1999, Lot and Chiang 1986, Miller and Nyberg 1995, Mitchell 1982, Robertson 1980, Smith 1971, Steenis 1977, Womersley 1981, Mori et al. 2016) as well as papers on the technical aspects of collecting specific groups of plants (Suppl. material 1; dol for all supplemental materail 10.6084/m9.figshare.4496981) so we will not cover detailed collecting methods in this manual.

## Voucher Specimen Collection

### What is a Voucher?

**Figure 1.**
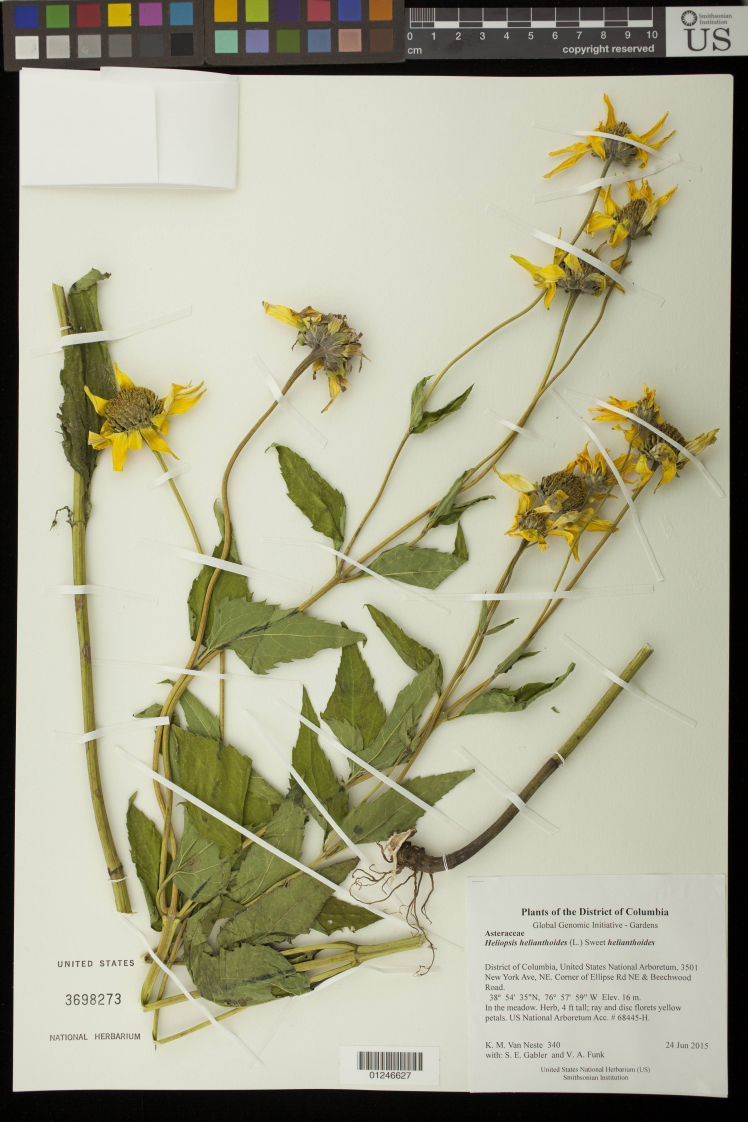
A “Specimen Voucher” from the US National Herbarium that is a dried, pressed herbarium voucher specimen with Catalog Number and specimen label. The collector + number is Van Neste 340, the sheet number is 3698273 and the specimen barcode is 01246627. doi for all figures 10.6084/m9.figshare.4496978

**Figure 2.**
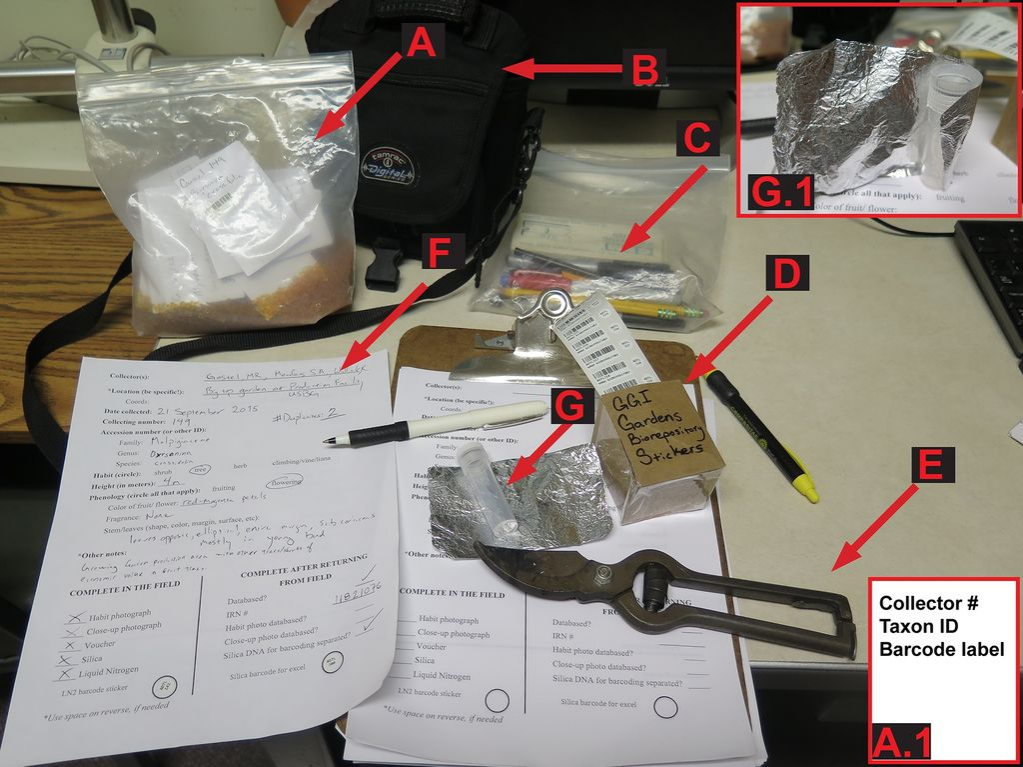
Example of a typical set of field equipment required for voucher specimen collection (for plant press and LN_2_ Dewar see Fig. 3). A. Bag with silica and envelopes, A1. Sample envelope, B. Camera, C. writing implements, D. Genomic sample barcode labels, E. clippers, F & F1. cryovial and aluminum foil, G & H. Data sheets. doi for all figures 10.6084/m9.figshare.4496978

**Figure 3.**
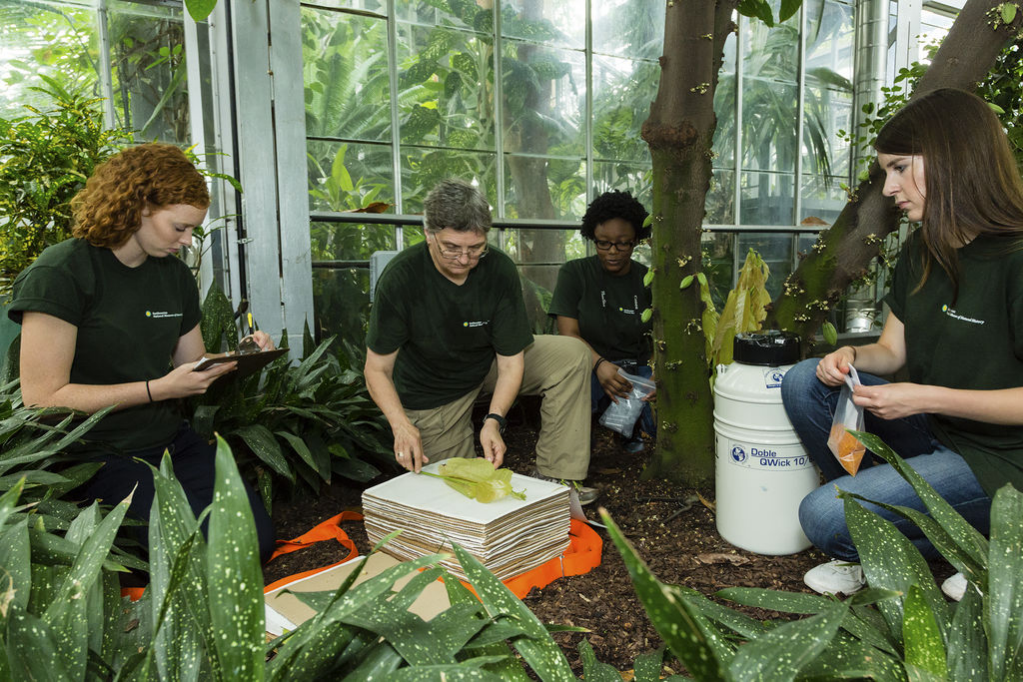
Example voucher specimen collection event with all necessary materials (from GGI–Gardens). From left to right: Sarah Gabler (summer undergraduate intern) is taking notes for the field book, V. Funk (Research Scientist, SI) is pressing the voucher specimen, Asia Hill (YES High School Intern) is preparing the tube for the liquid nitrogen sample, Kristen Van Neste (summer undergraduate intern) is waiting for leaf material to put into the coin envelope that will go into the orange silica gel in the reclosable polyethylene bag. [Photo courtesy of the Smithsonian Institution and GGI]. doi for all figures 10.6084/m9.figshare.4496978

If possible, all samples of tissue stored for genomic use should be associated with a voucher specimen. Usually a voucher is all or a portion of a plant that is collected, preserved, and maintained as reference material in an herbarium (Fig. 1). Since accurate species identification in the field is often impractical (e.g., an educated guess) and cultivated material is commonly misidentified, without a voucher it is often impossible to verify the correct name of a collection. A voucher specimen is typically used for taxonomic verification and morphological representation but can be used to infer a number of biological, ecological, and evolutionary characteristics about how and where the plant lived (Funk et al. 2015, Pleijel et al. 2008). The terms used to describe a voucher depend on the type of voucher and the relationship of the voucher to the genetic sample. The US National Herbarium (US) prefers pressed, dried, voucher specimens with reproductive material (Fig. 1), however, the type of voucher used depends on the materials at hand, and/or institutional protocol. An example of materials that should be brought into the field when collecting a voucher is shown in (Fig. 2) and a typical greenhouse collecting event is demonstrated in (Fig. 3).

### What information should be collected in your field notes as a part of the voucher?

Appropriate label information is as essential as the material collected for the voucher (see label in Fig. 1). For the labels, collectors should include details that are not intrinsic to the specimen. This information must include **biographic information** (collector, collecting number and any collecting team members), **biological data** (taxon name and/or family, character notes such as flower color, plant height, etc.), and **geographic data** (location, latitude x longitude). Latitude and longitude can be indicated in a number of ways but the best way to avoid confusion is to use decimal degrees. If possible one should include **ecological data** (e.g., soil type, landscape), and an indication that genetic samples were taken from the specimen. If more than one sample is to be mounted on a single sheet, then the individual that supplied the leaves for the genetic sample should be indicated. One method of identifying the voucher is to attach a tag to the specimen (Merchandise tags or Metal Rim tags are good options; Suppl. material 6, doi for all supplemental material 10.6084/m9.figshare.4496981). Genetic sample *unique identifying numbers* should be indicated in the field notebook and, if possible, on the label. It is also advisable to have a header on the label that indicates the collecting project (e.g., “Plants of the District of Columbia”) and a footer that lists the herbarium sponsoring the collecting trip (e.g. “Sponsored by the US National Herbarium & GGI-Gardens”). The format for entering these data may vary from collector to collector, but all collectors should have a clear, pre-determined organizational method for documenting this information, preferably in an archival field notebook that can be deposited in the herbarium library or other archival venue. In some instances the collector may prefer to fill out individual sheets for each collection and GGI-Gardens has sample collection sheets (Suppl. materials 2, 3; doi for all supplemental material 10.6084/m9.figshare.4496981). Plant collections from Gardens may have information indicating where the plant was obtained and these data should be requested from the Garden, GGI-Gardens has a data sheet for this type of information (Suppl. material 4; doi for all supplemental material 10.6084/m9.figshare.4496981) and when possible these data should be included on the label of the voucher specimen or on a separate label mounted on the voucher.

## Genetic Sample Collection

### Tissue Collections preserved in Silica Gel

**How to Choose and Work with Silica Gel**

**Figure 5.**
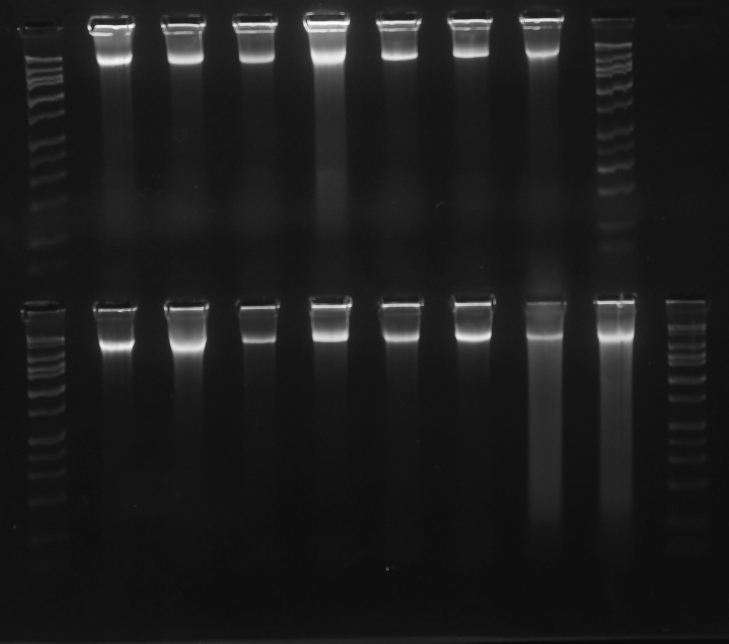
Gel image of high quality DNA (Vitaceae, ex J. Wen). DNA bands are tightly concentrated at the top of their lanes, with minimal streaking or blurring below. The HiLo gel ladder (Minnesota Molecular) at extreme left and right ranges from 50bp to 10,000bp. doi for all figures 10.6084/m9.figshare.4496978

Silica gel is used as a desiccant to rapidly dry recently collected plant tissues destined for DNA extraction (Fig. 5). For this reason, always store silica gel in a resealable (reclosable on some websites) air-tight container away from moisture (Suppl. material 610.6084/m9.figshare.449698; Fig. 3). A variety of silica gel types, as well as other kinds of desiccants (see below), have been successfully used by researchers. While finer grades (i.e. sand-like 28-200 mesh size) will dry tissues more quickly due to a greater surface area relative to volume, they are more difficult to work with and larger (less expensive) grades will typically dry adequately to get genomic quality DNA (Fig. 3). When working with all grades of silica gel, be aware of the hazards of accidental inhalation of silica dust, which can cause respiratory problems. Finer grades of gel can be more easily inhaled and use of a facemask is required (at US).

To assess the saturation of silica gel (to know when to change it out for fresh silica gel), indicating gel is used which undergoes a color change when saturated. The recommend ratio of indicating to non-indicating silica is usually 1:10. Indicators on the market come in 3 versions: **orange>>green** (based on methyl violet), **orange>>clear/white** (based on iron III/II salts), **blue>>pink** (based on cobalt (II) chloride). We recommend the more ‘environmentally friendly’ **orange>>clear/white**. Avoid using blue indicating gel as cobalt (II) chloride is an irritant, a carcinogen, and can cause environmental damage. Orange/green is potentially safer but not totally innocuous as the methyl violet is toxic and mutagenic. Orange/green is potentially safer but not totally innocuous as the methyl violet is toxic and mutagenic. An alternative, and perhaps better, choice over indicating gel, especially for bulk drying, is the use of a relative humidity indicator card which provides a semi-quantitative measure of relative humidity (RH) (Suppl. material 6, 10.6084/m9.figshare.4496981). They can be placed in each large resealable bag (made with inert salts instead of organic dyes). We are still investigating the cost and reusability of these cards.

To monitor moisture levels during storage, small amounts of indicator silica can be used or one can opt for *relative humidity indicator cards*; see Suppl. material 6). For storage, a relative humidity (RH) of 30% is a realistic target.

Once the plant material is dry the saturated silica gel can be exchanged for smaller amounts of fresh silica gel or a relative humidity indicator card (Fig. 3; see next section for more information). To save money the saturated silica gel may be reconditioned (reactivated) by heating to drive off water so that it is absorbent once more and can be reused (http://www.agmcontainer.com/desiccant-reactivator-unit.html). Do not put saturated silica gel in a forced-air plant dryer in an open container because silica dust can blow into the surrounding environment and may cause respiratory problems. In addition, some of the indicator gels are carcinogens. Instead put the silica gel into a cloth bag (e.g., pillowcase) before it goes into the plant dryer.

Alternative methods of desiccation include salt (Carrió and Rosselló 2013; P. Acevedo pers. com.), silica-gel cat litter (B. Brooks & A. Egan pers. com.), regular clay cat litter (A. Egan pers. com.) and even a food dehydrator (Tai and Tanksley 1990). Reports from the US National Herbarium Botany staff indicate that all of these substances work to dry leaf material. The clay based cat litter seems to be the least favorite because it takes longer to dry plant parts than the others. In the end, when available, most collectors prefer to dry their leaf material in silica.

**How to Preserve tissue Samples in Silica Gel**

When preserving plant material with silica gel, the goal is to gently dry samples as quickly as possible. The faster samples are dried, the higher the quality of DNA that can be extracted from them. Samples should be completely dried within 12–48 hours of collection, and should be dried in a cool, ambient-temperature environment (Chase and Hills 1991). When taking plant tissue for genetic samples, it is best to choose young (but not the very youngest) undamaged leaves. Young leaves have a higher density of cells per volume and should yield more DNA. However, the very youngest leaves are fragile and prone to damage before drying and the results are better with slightly older leaves. In general, older leaves are also more likely to have contaminants such as fungi or epiphylls. In wet tropical environments fungal endophytes rapidly colonize new leaves, especially from spores landing on those leaves (A. Herre, pers. comm.). Collect an amount of tissue that is approximately 10–25 cm2. Avoid stacking leaves in many layers because the inner leaves will dry more slowly than the outer ones. Leaves that are particularly thick, tough, or waxy should be torn (or cut) into small pieces (ca. 2 cm diameter) to increase the amount of exposed edge to draw water through. Clean the cutting tool and hands (hand sanitizer or wipes; gloves for critical samples) between samples to prevent cross contamination. If the leaves are dirty or contain epiphylls, wash in high quality water and pat dry. Other sources of DNA include flowers (corollas); which can be low in secondary compounds, and seeds. If preservation is delayed the tissues should be kept cool and moist to prevent wilting until they can be processed. Cold shock and deterioration (i.e., watery breakdown of tissues similar to freeze damage) can occur during refrigerator storage of sensitive species and may affect DNA quality. At the present time, we recommend collecting genetic samples directly into paper envelopes (2¼ x 3½ inches) such as ULINE S-11485, or small resealable polyethylene bags (2¼ x 3½ inches) such as ULINE® or Ziploc® (Suppl. material 6). The coin envelopes are porous, easy to label, allow for rapid drying of tissue samples, and are sized to fit into permanent storage boxes (such as Lock n Lock®) in the NMNH Biorepository, so little post-collecting processing work will need to be done. The coin envelopes are bleached, white paper envelopes but not necessarily archival quality. We are currently looking for a cost effective, storage solution where the paper is unbuffered as well as free of acidic constituents or other impurities (alpha cellulose paper with a neutral pH and no alkaline reserve, CAMEO 2016). The *Library of Congress* defines this type of paper on their website (accessed 2016) and the website sponsored by *The Conservation and Art Materials Encyclopedia Online* has additional information on Alpha cellulose (CAMEO 2016). Do not use brown or glassine envelopes that are acidic and less porous (respectively). The envelopes should be labeled with the primary collectors name and number. Before the envelopes are placed in the freezer for long-term storage they can be closed with archival paper clips (e.g., stainless steel; Suppl. material 6): while this is not necessary it can help keep the envelopes from overlapping with one another and prevent fragment escape. Envelopes should be placed in an airtight container or Ziploc® style bag and surrounded by silica gel to maximize surface area contact for drying (Fig. 3). Different sources estimate the correct amount of silica gel as anywhere from 9–15 times as much silica gel as plant matter and this partly depends on the water content of the tissues (Chase and Hills 1991). The silica gel should be gently mixed around the envelopes to prevent local saturation of the silica and examined several times a day to make sure the silica gel is not changing color. The benefit to storing tissue in separate envelopes is that you can place many samples together in one container (Fig. 2). Replacing hydrated silica gel in a shared container is less time consuming and the silica gel can be reused. If we are working out of an herbarium with a plant dryer we put the used silica into a large cotton bag (e.g., pillow case) and flatten it on the wire rack or shelf of the plant dryer and turn the temperature on high. In more remote areas it can be cooked in a large pot on a stove or over a fire. Care should be taken not to scorch the desiccant as this can cause a loss of moisture capacity and breakdown of the indicator. When the indicator has returned to its original color it is ready to be used again. There are alternative collection methods. For instance, instead of collecting into coin envelopes, some researchers collect into teabags or coffee filters that are very porous and allow samples to dry quickly. The drawbacks of teabags are that they are not as structured, so dried samples may be crushed more easily, and they are harder to label. For storage in the Biorepository, the collector may have to fold teabags and put them into coin envelopes or the samples may need to be transferred, resulting in extra processing time and/or excessive use of limited freezer space. Another method is to collect into small resealable polyethylene bags containing individual quantities of silica gel. The benefit is that plastic bags are easy to label, tissues easy to see, and because tissues are in direct contact with silica gel they can dry more quickly. When using polyethylene bags it is advisable to remove the silica on a rolling basis as samples dry. Dry samples can then be grouped in larger 1 gallon resealable bags. There are several drawbacks to the use of polyethylene bags. First, the dried leaves may be pulverized by the abrasive action of silica gel as samples are transported (making them harder to prepare in the lab). Second, it is extremely labor intensive when one needs to remove silica gel from each small bag. Third, the collector will need to empty the polyethylene bags of silica or transfer the samples to coin envelopes prior to storage in the Biorepository. Finally, it is more expensive because the silica is probably contaminated (leaf fragments, etc.) and should not be used again.

### Liquid Nitrogen Tissue Collection

For collection into liquid nitrogen, plant tissue samples are presently collected and placed into 8 ml, externally threaded cryovials with o-ring seals (Fig. 2). Tubes are labeled with a Biorepository barcode sticker (Fig. 2), wrapped in a ca. 4x8 cm piece aluminum foil (Fig. 2), and placed into a cryogenic storage Dewar filled with liquid nitrogen (Fig. 3). Samples begin to degrade immediately after collection, so it is important to work quickly. The foil is critical as the jostling of the tubes in the Dewar may cause the barcode stickers to dislodge or the caps to come off especially when the tubes are first put into the Dewar. Because the barcode sticker is the link between the collecting information and the sample it is critical that it be protected. Writing the collection number on the tube is a way to create a secondary identifier.

If LN2 is not available in the field, but LN2 collections are desired, one possibility is to bring germplasm (seeds, cuttings, plants) back from the field to grow locally. Genetic tissue samples could be cut from growing plants and deposited directly into LN2, and new voucher specimens collected directly from growing plants. Although it is labor and time-intensive, it provides excellent material under controlled conditions and for critical samples it is a viable alternative to working with LN2 at a remote collecting site.

### Ethanol Tissue Collection

Historically, ethanol has been used to preserve animal tissues for DNA studies but not plant tissues. There has been some investigation in the use of ethanol for plant tissue preservation (Flournoy et al. 1996). Preliminary results from a recent study suggest that, for some species, ethanol preservation may result in a higher yield of DNA but the quality of the recovered DNA is lower when compared to that recovered from the silica-dried method. The mechanism of preservation in alcohol is presumably dehydration, coupled with useful leaching of secondary compounds. As in animal tissues, higher alcohol concentrations (95–100%) appear to promote better preservation; changing the alcohol after an initial fixation can help reduce the total water for high water content plant tissues. The long-term viability of the DNA stored in ethanol has not been determined, however, preliminary tests show that while the quality of the immediately extracted DNA is excellent, the leaf material left in alcohol at room temperature for over a month is no longer superior to silica collected material (J. Wen, pers. comm.). The impact of temperature on degradation has not been studied in plants, but extrapolating from with animals work, cold storage of such alcohol collections will likely retard deterioration. For the storing of samples for genomic use we recommend silica or liquid nitrogen.

### RNA Collection and Preservation

**General precautions**

Tissues collected for RNA extraction must be handled with additional care in order to prevent the activity of RNases, both endogenous and those introduced by environmental contamination. This is not trivial as RNases are nearly ubiquitous, sturdy enzymes that are hard to “destroy”. When working with these tissues, wear disposable gloves and use disposable forceps or treat them with RNase-Away. If the goal is to preserve the transcriptome, it is important to process the sample as quickly as possible to prevent changes due to altered physiological state after collecting. While plants are slow to die after being picked, changes in gene expression can occur much faster and it is best to get tissues into the preservation medium quickly after harvesting (within seconds to a few minutes). If field decontamination methods are needed working surfaces can be cleaned by flaming with a micro-torch, dipping/wiping in chloroform (evaporates without residue but is carcinogenic), or a commercial decontamination solution (RNaseZap, RNase Away; but needs an RNase-free water wash afterwards). RNA can also be preserved by flash-freezing or special preservative solutions such as RNAlater®.

*RNAlater*® is specified in its patent (US 6528641) as a solution “composed of 25 mM sodium citrate, 10 mM EDTA, 70 g ammonium sulfate/100 ml solution, pH 5.2”, although subsequent patents have suggested improvements on this basic recipe. Pre-prepared tubes of RNAlater® are the easiest method of preserving RNA. Leaf tissues should be cut into small pieces (≤0.5 cm2 in any single dimension) and 5-10 volumes of RNAlater® to allow complete penetration. One problem with plant tissues is that they typically wet poorly in RNAlater® and float. To mitigate this issue use small vials that can be filled completely so that tissue can stay submerged, or macerate submerged tissue with a scalpel. Once tissues are in RNAlater® they should sit at room temperature to 4°C for 12–24 hours, then frozen (-20°C or -80°C). While manufacturer guidelines indicate room temperature storage for up to a week will not affect quality (or up to a month at 4°C), in general, colder storage sooner is better. Long-term storage at -80°C is recommended. RNAlater® will freeze into an opaque white block at -80°C and must be thawed to remove tissues; preferably most RNAlater® is poured off prior to -80°C storage, which will allow selection of samples without thawing.

Standard LN2 (see above) is another effective preservation method for RNA but keep in mind the comments above concerning the time between the removal of the tissue from the plant and its preservation.

## Samples in the NMNH Biorepository

Below, we discuss standard Biorepository specimen deposition methods. These methods are the most efficient and productive for long-term storage, databasing, sampling (genomic DNA and RNA). As always, specimen collection handling procedures should be performed with utmost care to ensure that the Biorepository collections are preserved according to the indefinite access and preservation goal of the Biorepository.

### Storage of silica preserved collections in the Biorepository

Silica dried samples are stored in -80°C freezers in the Biorepository. The current setup is storage of databased, tissue-filled coin envelopes in Lock & Lock brand plastic boxes (HPL836), which are airtight with a clamping lid and gasket. Each box has a custom plastic grid inside, producing 6–9 compartments where envelopes or bags are stored upright. The grids are adjustable to fit both the standard coin envelopes and the slightly larger bags that have been in use in Botany (Suppl. material 6). Within each box there is a packet of desiccant to keep samples as dry as possible and absorb moisture when boxes are opened. Boxes also have a relative humidity card to monitor the dryness.

### Storage of liquid nitrogen preserved collections in the Biorepository

Samples selected for LN2 preservation are collected into 8 ml cryovials. These cryovials will be stored in 3”, 6x6 gridded fiberboard boxes in vertical metal racks in the Biorepository liquid nitrogen freezers. When returning from field, as soon as possible, be sure to take all liquid nitrogen samples to the Museum Support Center (MSC) to be deposited in the Biorepository.

Please try to follow the recommended collection methods (collection into coin envelopes and 8 ml cryovials). By setting forth these specific collection methods, we are minimizing the amount of processing and rehousing work that will need to be done to incorporate samples into the Biorepository. This would be most efficient (rehousing is a very time consuming process) and would protect the integrity of the samples the best (less risk of contamination, less physical handling of delicate dried samples, etc.).

## Field Collecting Trips

### Collecting Event Approval/ Permits

It is strongly encouraged that all collecting permits be acquired prior to departure. In some countries it is not possible to obtain a collecting permit before you arrive but the application process for a permit should be well underway before the collector departs. In addition, if you intend to bring or ship plants into the USA you need an APHIS permit.

Collectors planning to work in foreign countries should be cognizant of the Nagoya Protocol (The Nagoya Protocol on Access to Genetic Resources and the Fair and Equitable Sharing of Benefits Arising from their Utilization to the Convention on Biological Diversity) that has been ratified by 92 countries (but not the USA) and went into effect on 12 October 2014. Under the Nagoya Protocol there are obligations to share benefits based on the utilization of genetic resources and there is a requirement for potential users to seek Prior Informed Consent (PIC) and to negotiate Mutually Agreed Terms (MAT) with governments and local indigenous peoples that hold traditional knowledge associated with the genetic resources (Schindel et al. 2015). Some countries have standardized forms that must be signed before collecting permits will be issued. The collector needs to check with others who have recently collected in the country to make sure of the details. The easiest way to navigate the new regulations is to work with a local collaborator who can participate in the fieldwork and research and guide you through the permitting process.

Some of the information below is specific to the US National herbarium (US), however, it should be useful as a guide to what can be put in place at other institutions. Specifically the text of this document is available in a Word format and the the FIMS spread sheet is available in Excel.

### For GGI partners

All GGI collections need to be databased, available on line, and deposited in a GGBN facility. Non-NMNH affiliated collectors should consult their home institution for database and/or collections database procedures.

## Supplementary Material

Supplementary material 1 Two lists of articles discussing various herbarium procedures and collecting techniques Data type: reference list
File: oo_116035.pdf Vicki Funk, Morgan Gostel, Amanda Devine, Carol Kelloff, Kenneth Wurdack, Chris Tuccinardi, Aleks Radosavljevic, Melinda Peters, Jonathan Coddington

Supplementary material 2 Example collecting sheet (from the GGI–Gardens project)showing preferred data entry fields included as part of a voucher specimen collection event Data type: collecting sheet
File: oo_116036.pdf Vicki Funk, Morgan Gostel, Amanda Devine, Carol Kelloff, Kenneth Wurdack, Chris Tuccinardi, Aleks Radosavljevic, Melinda Peters, Jonathan Coddington

Supplementary material 3 Alternative collecting sheet showing data entry fields forelectronically entered Genetic Sample data Data type: collecting sheet
File: oo_116037.pdf Vicki Funk, Morgan Gostel, Amanda Devine, Carol Kelloff, Kenneth Wurdack, Chris Tuccinardi, Aleks Radosavljevic, Melinda Peters, Jonathan Coddington

Supplementary material 4 Provenance for Garden Plants: Original Collection Information found on voucher label or from Garden database Data type: collection information
File: oo_116038.pdf Vicki Funk, Morgan Gostel, Amanda Devine, Carol Kelloff, Kenneth Wurdack, Chris Tuccinardi, Aleks Radosavljevic, Melinda Peters, Jonathan Coddington

Supplementary material 5 Making a Plant Specimen Dryer Data type: guide, images
File: oo_116039.pdf Carol Kelloff

Supplementary material 6 Sources for commonly used resources Data type: refrence and sources list
File: oo_116040.pdf Vicki Funk, Morgan Gostel, Amanda Devine, Carol Kelloff, Kenneth Wurdack, Chris Tuccinardi, Aleks Radosavljevic, Melinda Peters, Jonathan Coddington

## Figures and Tables

**Figure 4a.**
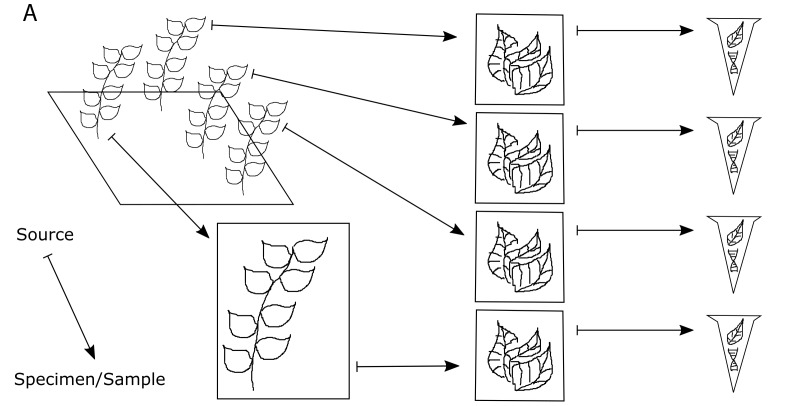
One voucher specimen and three genetic (tissue) samples are taken from an individual in the field. In addition, one genetic (tissue) sample is taken from the voucher specimen. Genetic (DNA) samples are extracted from each of the genetic (tissue) samples.

**Figure 4b.**
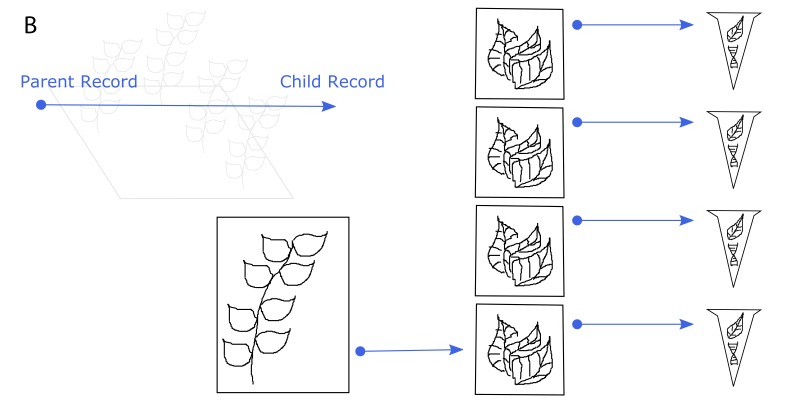
Every sample that is derived from another specimen/sample is a child of that specimen/sample. The original individual in the field is not cataloged, so it is not a parent. The bottommost DNA extract is a child of the bottommost tissue sample, which is a child of the voucher specimen.

**Figure 4c.**
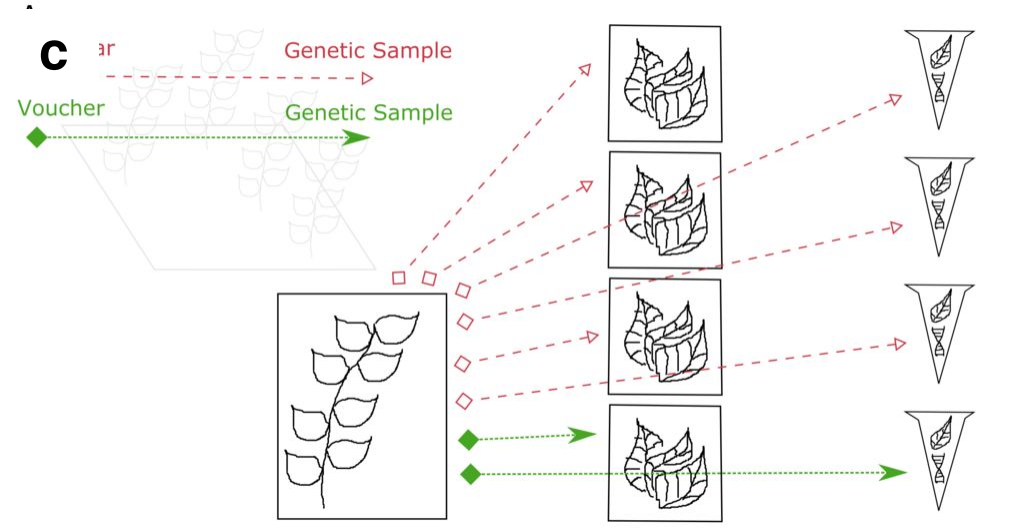
Illustration of the Voucher and Exemplar designations. When genetic samples are taken directly from the pressed, dried specimen, that specimen is the voucher of those genetic samples. When genetic samples are taken from individuals that are different than the pressed, dried specimen, but are thought to be the same species, then the pressed, dried specimen is the exemplar of those genetic samples.

**Figure 6.**
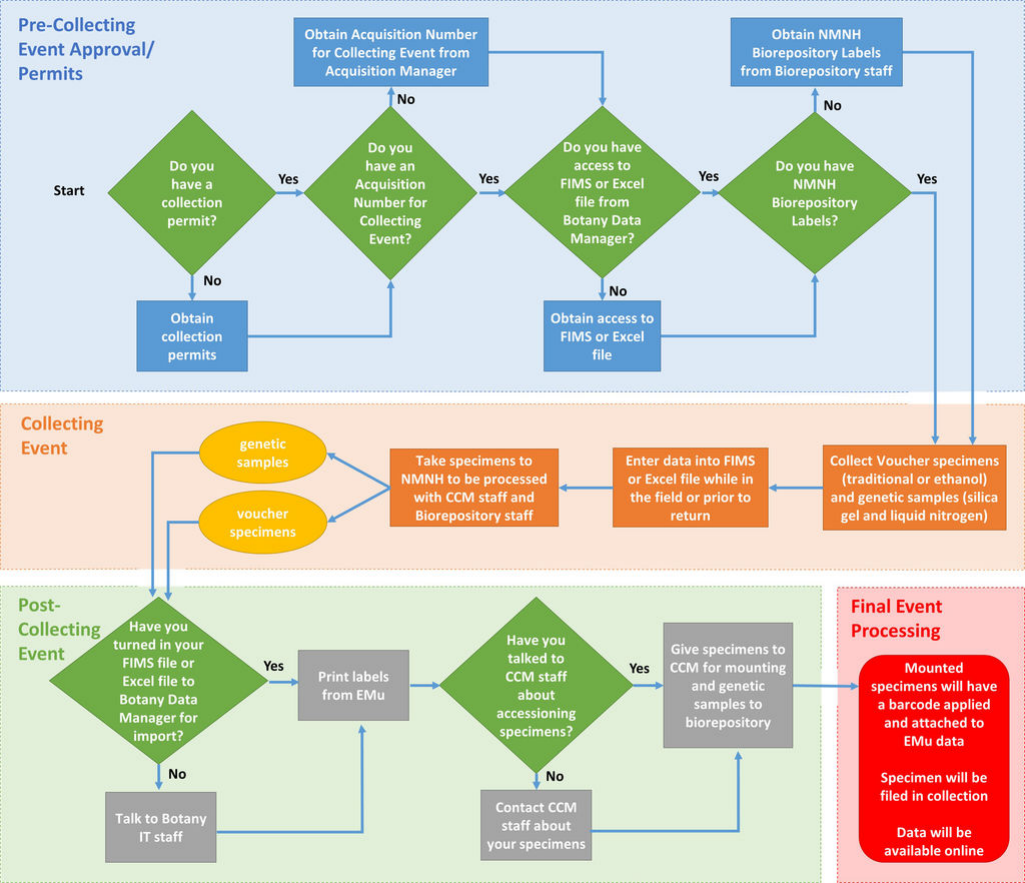
Flow Chart illustrating the GGI-Gardens sampling strategy. Extracted from a poster entitled “Incorporating Genetic Sampling into a Traditional Botanical Voucher Workflow” presented at the GGBN meeting in Berlin, June 2016, by Melinda Peters and Amanda M. Devine (US). doi for all figures 10.6084/m9.figshare.4496978
