# Supplementary material for: Guidelines for collecting vouchers and tissues intended for genomic work (Smithsonian Institution): Botany Best Practices
Source: Biodivers Data J. 2017 Jan 30;(5):e11625. doi: 10.3897/BDJ.5.e11625 (PMC5345056; doi:10.3897/BDJ.5.e11625)
Supplement: Supplementary material 1 — Two lists of articles discussing various herbarium procedures and collecting techniques [file bdj-05-e11625-s001.pdf]

Supplemental Material 1: Two lists of articles discussing various herbarium procedures and collecting techniques

List 1: Publications organized by Taxon or Group

Algae: González-González J, Novelo-Maldonado E (1986) Algas. Pages 47–54, in Lot A, Chiang F (eds) Manual de Herbaria: Administraction y Manejo de Collectiones Tecnias de Recoleccion y Preparacion de Ejemplares Botanicos. National Council of the Flora of Mexico: Mexico.
https://drive.google.com/file/d/0BwQw3z-V2CP3U3h4eUtkZ2wtQVk/view

Algae: Sims RS (2016) Algae Research: Collection and Preservation.
http://botany.si.edu/projects/algae/collpres.htm

Aquatics: Ceska A, Ceska O (1986) More on techniques for collecting aquatic and marsh plants. Annals of the Missouri Botanical Garden 73(4): 825-827.
http://www.biodiversitylibrary.org/item/87377#page/839/mode/1up

Aquatics: Fosberg FR, Sachet M-H (1965) Instructions for collecting macroscopic aquatic plants. Pages 107–109, in Fosberg FR, Sachet H-M, Manual for Tropical Herbaria. Regnum Vegetabile 39, International Bureau for Plant Taxonomy and Nomenclature, Utrecht. [Republished in JS Womersley (1981) Plant collecting and herbarium development: a manual. Pp 98–99. Food and Agriculture Organization of the United Nations: Rome]

Aquatics: Haynes RR (1984) Techniques for collecting aquatic and marsh plants. Annals of the Missouri Botanical Garden 71: 229–231.
http://www.biodiversitylibrary.org/item/54734#page/242/mode/1up

Aquatics: Lot A (1986) Aquáticas vasculares. In: Lot A, Chiang F (eds) Manual de Herbaria: Administraction y Manejo de Collectiones Tecnias de Recoleccion y Preparacion de Ejemplares Botanicos. Pp 87–92. Mexico: National Council of the Flora of Mexico.
https://drive.google.com/file/d/0BwQw3z-V2CP3U3h4eUtkZ2wtQVk/view

Aquatics: Saunders GW, Daniel CM (2012) Chapter 10, Methods for DNA Barcoding Photosynthetic Protists Emphasizing the Macroalgae and Diatoms. Pages 207–222 in, Kress WJ, Erickson DL (eds.), DNA Barcodes: Methods and Protocols, Methods in Molecular Biology, vol. 858, DOI 10.1007/978-1-61779-591-6_10.

Araceae: Nicolson DH (1965) Collecting Araceae. In: Fosberg, F.R. and M.-H. Sachet (eds) Manual for Tropical Herbaria. Regnum Vegetabile 39. pp. 123–126. International Bureau for Plant Taxonomy and Nomenclature, Utrecht.

Araceae: Nicolson DH (1981) Notes on the collection of Araceae. Pages 115–120 in JS Womersley, Plant collecting and herbarium development: a manual. Food and Agriculture Organization of the United Nations: Rome [updated from Nicolson 1965]

Araceae: Croat, TB (1985) Collecting and preparing specimens of Araceae. Annals of the Missouri Botanical Garden 72(2): 252–258.
http://www.biodiversitylibrary.org/item/89033#page/262/mode/1up

Balsaminaceae, Impatiens: Grey-Wilson C (1980) Notes on collecting Impatiens. Flora Malesiana Bulletin 33: 3435–3436.

Begoniaceae, Begonia: Logan J (1986) A pre-pressing treatment for Begonia species and succulents. Taxon 35: 671.

Bromeliaceae: Jorgensen V (1972) The preparing, pressing and mounting of bromeliads. Journal of the Bromeliad Society 23: 211–214.

Bryophytes: Delgadillo C (1986) Briófitas. Pages 77–82 In: A Lot, Chiang F (eds) Manual de Herbaria: Administraction y Manejo de Collectiones Tecnias de Recoleccion y Preparacion de Ejemplares Botanicos. National Council of the Flora of Mexico: Mexico.
https://drive.google.com/file/d/0BwQw3z-V2CP3U3h4eUtkZ2wtQVk/view

Cyclanthaceae: Hammel BE (1987) The origami of Botany: A guide to collecting and mounting specimens of Cyclanthaceae. Annals of the Missouri Botanical Garden 74(4): 897–902.
http://www.biodiversitylibrary.org/item/87376#page/913/mode/1up

Epiphytes: Aguirre-León E (1986) Epiphytes [including Orchidaceae]. Pages 113–119 Lot A & F Chiang (1986) Manual de Herbario: Administracion y manejo de colecciones, tecnicas de recoleccion y preparacion de ejemplares botanicos. Mexico: National Council of the Flora of Mexico. [Orchidaceae, Bromeliaceae]
https://drive.google.com/file/d/0BwQw3z-V2CP3U3h4eUtkZ2wtQVk/view

Ferns: Arreguín-Sánchez M de la L (1986) Pteridófitas. Pages 83–86, in A Lot & F Chiang (eds) Manual de Herbaria: Administraction y Manejo de Collectiones Tecnias de Recoleccion y Preparacion de Ejemplares Botanicos. National Council of the Flora of Mexico: Mexico.
https://drive.google.com/file/d/0BwQw3z-V2CP3U3h4eUtkZ2wtQVk/view

Ferns: Brownsey PJ (1985) A plea for better collecting and curation of large ferns. Newsletter of the Australian Systematic Botany Society 43: 17–19.

Ferns: Croft J (1999) A guide to collecting herbarium specimens of ferns and their allies. [accessed 3 June 2016] http://www.anbg.gov.au/fern/collecting.html

Ferns: Holttum RE (1957) Instructions for collecting tree ferns. Flora Malesia Bulletin 13: 567.

Ferns: Janssen T (2006) Moulding method to preserve tree fern trunk surfaces including remarks on the composition of tree fern herbarium specimens. Fern Gazette 17(6,7,8): 283–295

Ferns: Stolze RG 1973. Inadequacies in herbarium specimens of large ferns. American Fern Journal 63: 25–27.

Fungi: Cifuentes-Blanco J, Villegas Ríos M, Lérez-Ramírez L (1986) Hongos. Pages 55–64, in A Lot, Chiang F (eds) Manual de Herbaria: Administraction y Manejo de Collectiones Tecnias de Recoleccion y Preparacion de Ejemplares Botanicos. National Council of the Flora of Mexico: Mexico. https://drive.google.com/file/d/0BwQw3z-V2CP3U3h4eUtkZ2wtQVk/view

Lecythidaceae: Mori SA, Prance GT (1987) A guide to collecting Lecythidaceae. Annals of the Missouri Botanical Garden 74(2): 321–330.

Lentibulariaceae Utricularia: Taylor P, Steenis C van (1977) On the collection and preparation of Utricularia specimens. Flora Malesiana Bulletin 30: 2831–2832

Lianas: Gerwing, JJ, Schnitzer SA, Burnham RJ, Bongers F, Chave J, DeWalt SJ, Ewango CEN, Foster R, Kenfack D, Martínez-Ramos M, Parren M, Parthasarathy N, Pérez-Salicrup DR, Putz FE, Thomas DW (2006) A standard protocol for liana censuses. Biotropica 38(2): 256–261.

Lichens: Coutiño B (1986) Líquenes. Pages 65–75, in A Lot & F Chiang (eds) Manual de Herbaria: Administraction y Manejo de Collectiones Tecnias de Recoleccion y Preparacion de Ejemplares Botanicos. National Council of the Flora of Mexico: Mexico. https://drive.google.com/file/d/0BwQw3z-V2CP3U3h4eUtkZ2wtQVk/view

Molecular samples: Neubig KM, Whitten WM, Abbott JR, Elliott S, Soltis DE, Soltis PS (2014) Variables affecting DNA preservation in archival DNA specimens. Pages 81–136, in Applequist WL & LM Campbell, DNA Banking for the 21st Century: Proceedings of the U.S. Workshop on DNA banking. Missouri Botanical Garden: St Louis.

Molecular samples: Spooner DM, Ruess H (2014) Curating DNA specimens. Pages 71–80 in Applequist WL & LM Campbell, DNA Banking for the 21st Century: Proceedings of the U.S. Workshop on DNA banking. Missouri Botanical Garden: St Louis.

Musaceae, bananas: Womersley JS (1981) Instructions for collecting botanical specimens of banana (Musa) plants. Pages 100–102, in Womersley JS, Plant collecting and herbarium

development: a manual. Food and Agriculture Organization of the United Nations: Rome. Pp 137

Palms: Balick, MJ (1989) Collection and preservation of palm specimens. Pages 482–483 in: Campbell DG, Hammond HD (eds), Floristic Inventory of Tropical Countries. New York Botanical Garden, New York.

Palms: Dransfield J (1979) A manual of the rattans of the Malay Peninsula. Malayan Forest Records 29: 1–270.

Palms: Dransfield, J (1986) A guide to collecting palms. Annals of the Missouri Botanical Garden 73(1) 166–176. http://www.jstor.org/stable/2399148?seq=1#page_scan_tab_contents

Palms: Quero HJ (1986) Palmas. Pages 121–131, in A Lot, Chiang F (eds) Manual de Herbaria: Administraction y Manejo de Collectiones Tecnias de Recoleccion y Preparacion de Ejemplares Botanicos. National Council of the Flora of Mexico: Mexico. https://drive.google.com/file/d/0BwQw3z-V2CP3U3h4eUtkZ2wtQVk/view

Palms: Tomlinson PB (1965) Special techniques for collecting palms for taxonomic study. In: FR Fosberg & M-H Sachet (eds) Manual for Tropical Herbaria. Regnum Vegetabile 39. pp.112–116. International Bureau for Plant Taxonomy and Nomenclature, Utrecht [updated by Fosberg in Womersley JS 1981, pages 103–109].

Pandanaceae: Stone BC (1981) Instructions for collecting botanical specimens of Pandanus (pandans). Pages 94–97, in Womersley JS, Plant collecting and herbarium development: a manual. Food and Agriculture Organization of the United Nations: Rome.

Pandanaceae: Stone BC (1983) A guide to collecting Pandanaceae (Pandanus, Greycinetia, and Sararanga). Annals of the Missouri Botanical Garden 70(1): 137–145. http://www.biodiversitylibrary.org/item/54746#page/145/mode/1up

Passifloraceae, Passiflora: Jorgensen P, Lawesson JE, Holm-Nielsen LB (1984) A guide to collecting passionflowers. Annals of the Missouri Botanical Garden 71(4): 1172–1174. http://www.biodiversitylibrary.org/item/54734#page/1195/mode/1up

Poaceae, Bamboo: Soderstrom T, Young SM (1983) A guide to collecting bamboos. Annals of the Missouri Botanical Garden 70(1): 128–136. http://www.biodiversitylibrary.org/item/54746#page/136/mode/1up

Poaceae, Bamboo: McClure FA (1965) Suggestions on how to collect bamboos. Pages 121–122 in: Fosberg FR & M-H Sachet (eds) Manual for Tropical Herbaria. Regnum Vegetabile 39. International Bureau for Plant Taxonomy and Nomenclature, Utrecht.

Poaceae, Bamboo: McClure FA (1981) Notes on the collection of bamboos for identification and taxonomic study. Pages 110–114 in, Womersley JS, Plant collecting and herbarium development: a manual. Food and Agriculture Organization of the United Nations: Rome [updated from McClure 1965]

Poaceae: Koch SD (1986) Gramíneas y graminoides. Pages 93–101 in Lot A, Chiang F, Manual de Herbario: Administracion y manejo de colecciones, tecnicas de recoleccion y preparacion de ejemplares botanicos. Mexico: National Council of the Flora of Mexico. National Council of the Flora of Mexico: Mexico.

Pollen: Le Thomas, A (1989) Collection and preparation of pollen samples. In: Campbell DG, Hammond HD (eds) Floristic Inventory of Tropical Countries. pp. 474–475. New York Botanical Garden, New York

Succulents: Baker M, Mohlenbrock MW, Pinkava DJ (1985) A Comparison of Two New Methods of Preparing Cacti and Other Stem Succulents for Standard Herbarium Mounting. Taxon 34(1): 118–120.

Succulents: Snchez-Mejorada H (1986) Suculentas. Pp. 103–111, in Lot A, Chiang F, Manual de Herbario: Administracion y manejo de colecciones, tecnicas de recoleccion y preparacion de ejemplares botanicos. Mexico: National Council of the Flora of Mexico. [includes Agavaceae, Cactaceae, Crassulaceae]
https://drive.google.com/file/d/0BwQw3z-V2CP3U3h4eUtkZ2wtQVk/view

Trees: Wendt T (1986) Arboles. Pages 133–142m, in: Lot, A & F Chiang (eds) Manual de Herbaria: Administraction y Manejo de Collectiones Tecnias de Recoleccion y Preparacion de Ejemplares Botanicos. Pp 133–142. Mexico: National Council of the Flora of Mexico.
https://drive.google.com/file/d/0BwQw3z-V2CP3U3h4eUtkZ2wtQVk/view

Wood: Womersley JS (1981) Collection of wood samples. Pages 121–122 in Plant collecting and herbarium development: a manual. Food and Agriculture Organization of the United Nations: Rome.

Zingiberaceae: Burtt BL, Smith RM (1976) Notes on the collection of Zingiberaceae. Flora Malesiana Bulletin 29(1): 2599–2600. http://repository.naturalis.nl/record/533127

List 2: General Publications on Collecting Techniques

Applequist WL, Campbell LM (2014) DNA Banking for the 21st Century: Proceedings of the U.S. Workshop on DNA banking. Missouri Botanical Garden: St Louis. 187 Pages

Davis AP (2011) Chapter 27 Collecting herbarium vouchers. In Collecting Plant Diversity; Technical Guidelines, L. Guarino, V. Ramanatha Rao & E. Goldberg (eds.) published by Bioversity International. [general discussion of collecting vouchers] http://cropgenebank.sgrp.cgiar.org/index.php?option=com_content&view=article&id=663

Davis PH (1961) Hints for hard-pressed collectors. Watsonia 4(6): 283–289 http://www.biodiversitylibrary.org/bibliography/83809#/summary

Fosberg FR, Sachet M-H (1965) Manual for Tropical Herbaria. Regnum Vegetabile 39, pages 1–132. International Bureau for Plant Taxonomy and Nomenclature, Utrecht. Pp 1–132. [macroscopic aquatic plants; bananas; fungi; palms; bamboo; Araceae; succulents; wood samples]

Hyland RPM (1972) A technique for collecting botanical specimens in rainforests. Flora Malesiana Bulletin 26: 2038–2040.

Jain SK, Mudgal V (1999) A Hand Book of Ethnobotany. Publisher: Bishen Singh Mahendra Pal Singh. 322 pages

Lot A, Chiang F (1986) Manual de Herbario: Administracion y manejo de colecciones, tecnicas de recoleccion y preparacion de ejemplares botanicos. Mexico: National Council of the Flora of Mexico. 142 pages [algae, fungi, lichens, mosses, ferns, aquatic vascular plants, grasses, succulents, epiphytes, palms, trees] https://drive.google.com/file/d/0BwQw3z-V2CP3U3h4eUtkZ2wtQVk/view

Miller AG, Nyberg JA (1995) Chapter 27 Collecting herbarium vouchers. Pages 561–573, in Collecting Plant Diversity; Technical Guidelines, Guarino L, Ramanatha Rao V, Reid R (eds.). CAB International [for updated version see Davis 2011] http://cropgenebank.sgrp.cgiar.org/images/file/procedures/collecting1995/Chapter27.pdf

Mitchell AW (1982) Reaching the Rainforest Roof- A Handbook on Techniques of Access and Study in the Canopy. Leeds Philosophical and Literary Society and UNEP, Leeds

Robertson KR (1980) Observing, Photographing and Collecting Plants. Illinois Natural History Survey Circular 55, pages 1-72 + Cover page and Table of Contents. http://hdl.handle.net/2142/73357

Smith CE (1971) Preparing Herbarium Specimens of Vascular Plants. USDA Agricultural Information Bulletin No. 348.

Steenis CGGJ van (1977) Three pleas to collectors - improve your field data. Flora Malesiana Bulletin 30: 2843-2844.

Womersley JS (1981) Plant collecting and herbarium development: a manual. Food and Agriculture Organization of the United Nations: Rome. Pp 137 [pandans; aquatics; banana; palms; bamboos, Araceae; wood]
