# Supplementary material for: Guidelines for collecting vouchers and tissues intended for genomic work (Smithsonian Institution): Botany Best Practices
Source: Biodivers Data J. 2017 Jan 30;(5):e11625. doi: 10.3897/BDJ.5.e11625 (PMC5345056; doi:10.3897/BDJ.5.e11625)
Supplement: Supplementary material 2 — Example collecting sheet (from the GGI–Gardens project)showing preferred data entry fields included as part of a voucher specimen collection event [file bdj-05-e11625-s002.pdf]

Supplemental Material 2: Example collecting sheet (from the GGI–Gardens project) showing preferred data entry fields included as part of a voucher specimen collection event

Collector(s): ____

***Location (be specific!):** ____

Coords:

Date collected:

Collecting number: **Number of Duplicates:**

Accession number (or other ID):

Family:

Genus:

Species:

Habit (circle): shrub tree herb climbing/vine/liana

Height (in meters): _______

Phenology (circle all that apply): fruiting flowering

Color of fruit/ flower: ________

Fragrance:

Stem/leaves (shape, color, margin, surface, etc):

***Other notes:**

COMPLETE IN THE FIELD

_____ Habit photograph

_____ Close-up photograph

_____ Voucher

_____ Silica

_____ Liquid Nitrogen

LN2 barcode sticker ◯

COMPLETE AFTER RETURNING FROM FIELD

Databased? _____

IRN # ___________

Habit photo databased? _____

Close-up photo databased? _____

Silica DNA for barcoding separated? _____

Silica barcode for excel ◯

**Use space on reverse, if needed*
