# Supplementary material for: Guidelines for collecting vouchers and tissues intended for genomic work (Smithsonian Institution): Botany Best Practices
Source: Biodivers Data J. 2017 Jan 30;(5):e11625. doi: 10.3897/BDJ.5.e11625 (PMC5345056; doi:10.3897/BDJ.5.e11625)
Supplement: Supplementary material 3 — Alternative collecting sheet showing data entry fields forelectronically entered Genetic Sample data [file bdj-05-e11625-s003.pdf]

Supplemental Material 3: Alternative collecting sheet showing data entry fields for electronically entered Genetic Sample data

GGI–Gardens collection **Date:**

Collector	Collector Number	LN2 Sticker	Silica Sticker

Collector	Collector Number	LN2 Sticker	Silica Sticker
