# Supplementary material for: Guidelines for collecting vouchers and tissues intended for genomic work (Smithsonian Institution): Botany Best Practices
Source: Biodivers Data J. 2017 Jan 30;(5):e11625. doi: 10.3897/BDJ.5.e11625 (PMC5345056; doi:10.3897/BDJ.5.e11625)
Supplement: Supplementary material 4 — Provenance for Garden Plants: Original Collection Information found on voucher label or from Garden database [file bdj-05-e11625-s004.pdf]

Supplemental Material 4: Provenance for Garden Plants: Original Collection Information found on voucher label or from Garden database

Collector of the original material:

Collecting Number:

Family:

Subfamily:

Tribe:

Genus:

Species:

Authority:

Subspecific category:

Authority:

Original Collection date:

Country of Origin:

1st political division:

Location:

lat x long, elevation:

Habitat:

Plant description:

Notes:
