# Supplementary material for: Guidelines for collecting vouchers and tissues intended for genomic work (Smithsonian Institution): Botany Best Practices
Source: Biodivers Data J. 2017 Jan 30;(5):e11625. doi: 10.3897/BDJ.5.e11625 (PMC5345056; doi:10.3897/BDJ.5.e11625)
Supplement: Supplementary material 5 — Making a Plant Specimen Dryer [file bdj-05-e11625-s005.pdf]

Supplemental Material 5: Making a Plant Specimen Dryer
(http://botany.si.edu/bdg/plantdry/index.htm) by Carol Kelloff

Materials needed:
Angled slotted zinc can be purchased in any local hardware store. You will need at least 4 pieces of angled zinc 1-1/2" x 72".
16 - 1" bolts
16 - wing nuts
8" x 24" board
2 - 150 watt flood lamps
2 - lamp sockets
4 - wood screws
3 yards of canvas ca. 24" width or 1.5 yards of canvas if 48" in width
8 - 1" brass grommets
10 feet of 16/3 power cord with plug

Plant specimen dryer channels: Cut the zinc into 4 pieces 18-3/4" in length and 8 pieces 24" in length. Remember to cut the zinc so that the holes line up.

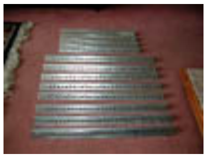

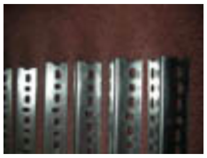

Plant specimen dryer lights: Cut one piece 16/3 power cable 10 inches long. Strip off the ends exposing the wires. Connect the two lamp sockets (in series). Strip the bare end of the 16/3 power cord with plug to expose the wires and connect to one lamp socket. Screw the lamp sockets to 8" x 24" board approx. 6 - 8 inches apart.

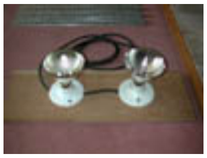

Plant specimen dryer bolts: These are the 16 1" bolts and 16 wing nuts. Pick a diameter (probably 3/8") that is right for the holes in your Zinc frame.

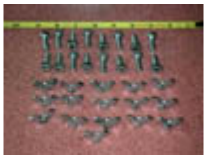

Plant specimen dryer with lights: The bottom will be used to rest the lamp board and the end zinc angles can be moved to adjust the height of the lamps.

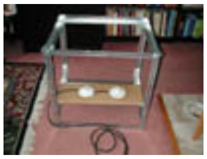

Plant dryer frame: Assemble the front and back framework of the dryer using four (4) 24" zinc per side. Fasten the zinc at the corners using the bolts with the wing nuts facing out. Place the angle of the zinc down, ie: **L**. This will provide a "shelf" for the lamp board and plant press to rest. For the sides use the 18-3/4" pieces of zinc.

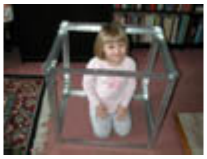

Plant specimen dryer finishing:

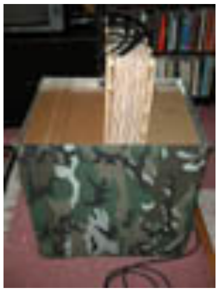

Cut the canvas 24" wide and approx. 88" in length. Fasten a brass grommet in the upper left corner of canvas and attach using wing nut and bolt of frame. Stretch canvas to next corner, place grommet appropriately and attach to frame. Keep going around frame until canvas is attached to frame. You may want to add a grommet to the bottom corners of canvas where the two ends meet. This will hold the canvas tight to the frame on the bottom.

A standard plant press will sit on the lower angle of the zinc at the top of the dryer. Close off any space on either side of a thin plant press by using a board or cardboard. Tighten and flip the press daily or as needed. Drying time varies according to several factors including specimen characteristics, humidity, and air circulation. Recheck specimens after they have been off of the dryer for a day or two to see if more drying is actually required.

CAUTION: FIRE HAZARD Check your plant drier frequently. Remember that you are dealing with paper and heat. Keep the heat source at a reasonable distance from the press. Make sure the press straps are not hanging on or near the heat source.

Any heat source, incandescent or involving combustion, can be **dangerous** and neither the author nor Smithsonian take any responsibility for losses or injuries.

Use common sense!
