# Supplementary material for: Guidelines for collecting vouchers and tissues intended for genomic work (Smithsonian Institution): Botany Best Practices
Source: Biodivers Data J. 2017 Jan 30;(5):e11625. doi: 10.3897/BDJ.5.e11625 (PMC5345056; doi:10.3897/BDJ.5.e11625)
Supplement: Supplementary material 6 — Sources for commonly used resources [file bdj-05-e11625-s006.pdf]

**Supplemental Material 6: Sources for commonly used resources**

**Indicator CARDS** for measuring moisture content
http://www.universityproducts.com/cart.php?m=product_list&c=1131&primary=1&parentId=&navTree%5b%5d=1131 [25 cents ea. (US$) if you order 25]
or
http://www.sorbentsystems.com/hic.html [14 cents ea. (US$) if you order 500]

**CRYOTUBES (8 ml)**
Sarstedt: https://www.sarstedt.com/en/home/
57x16.5 mm, polypropylene with EPDM o-ring, 8 ml sample volume; 60.542.024 (assembled and sterile); 60.542.007 (separate tubes and caps)

**Paper coin ENVELOPS**
ULINE S-11485
http://www.uline.com/Product/Detail/S-11485/Paper-Office-Envelopes/2-1-4-x-3-1-2-White-Coin-Envelopes

**Stainless Steel PAPER CLIPS (1.25 in, 3.2 cm)**
LINECO 497-2001
http://www.lineco.com/cart.php?m=product_list&c=536&s=1026

**Resealable (Reclosable) POLYETHYLENE BAGS**
http://www.uline.com/Grp_5/Poly-Bags-Reclosable
or Ziploc® from any grocery store

**Plant PRESS and other collecting gear**
Herbarium Supply Company
P.O. Box 10966
Bozeman, MT 59719 U.S.A.
http://herbariumsupply.com/product-category/pressing/presses/

**Bulk SILICA gel**
Sorbent Systems
http://www.sorbentsystems.com/bulksorbents.html
640SGO55, Orange indicating silica gel, 2-4mm bead, 55 lb drum
639AG55BG, White Silica Gel, 2-4mm bead, 55 lb drum
*or*
AGM Container Controls, Inc
http://www.agmcontainer.com/products/desiccants/bulk-desiccant.html
920010, Flower Dry Silica Gel, 0.08-0.6 mm bead (fine granular grade, blue indicator), 55 lb bag
920004, Silica gel mix, 90% white, 10% blue indicating, 2-5 mm bead, 55 lb bag
920013, Orange Indicating Silica Gel, 2-5 mm bead, 55 lb bag

**Plastic STORAGE BOXES used in the Biorepository**

Lock & Lock USA http://shop.locknlock-usa.com/   (sometimes out of stock) HPL836, 1.45 Gallon container
Also from: http://www.qvc.com/kitchen-&-food/lock-&-lock/_/N-lglvZ1z141jy/c.html?pageSize=ALL

**Paper TAGS: Merchandise tags (variety of colors and sizes) or METAL RIM tags** (variety of sizes) http://www.uline.com/BL_3009/Metal-Rim-Tags
